# Relationship between different serum cartilage biomarkers in the acute response to running and jumping in healthy male individuals

**DOI:** 10.1038/s41598-022-10310-z

**Published:** 2022-04-19

**Authors:** Maren Dreiner, Tobias Munk, Frank Zaucke, Anna-Maria Liphardt, Anja Niehoff

**Affiliations:** 1 Institute of Biomechanics and Orthopaedics, German Sport University Cologne, Am Sportpark Müngersdorf 6, 50933 Cologne, Germany; 2 Dr. Rolf M. Schwiete Research Unit for Osteoarthritis, Department of Orthopaedics (Friedrichsheim), University Hospital Frankfurt, Goethe University, Frankfurt, Germany; 3 Department of Internal Medicine 3 - Rheumatology and Immunology, Universitätsklinikum Erlangen and Friedrich-Alexander University Erlangen-Nürnberg, Erlangen, Germany; 4 Faculty of Medicine, Cologne Center for Musculoskeletal Biomechanics (CCMB), University of Cologne, Cologne, Germany

**Keywords:** Biomarkers, Molecular medicine

## Abstract

The effect of physical activity on serum cartilage biomarkers is largely unknown. The purpose of the study was to systematically analyze the acute effect of two frequently used exercise interventions (running and jumping) on the correlation of seven serum biomarkers that reflect cartilage extracellular matrix metabolism. Fifteen healthy male volunteers (26 ± 4 years, 181 ± 4 cm, 77 ± 6 kg) participated in the repeated measurement study. In session 1, the participants accomplished 15 × 15 series of reactive jumps within 30 min. In session 2, they ran on a treadmill (2.2 m/s) for 30 min. Before and after both exercise protocols, four blood samples were drawn separated by 30 min intervals. Serum concentrations of seven biomarkers were determined: COMP, MMP-3, MMP-9, YKL-40, resistin, Coll2-1 and Coll2-1 NO_2_. All biomarkers demonstrated an acute response to mechanical loading. Both the COMP and MMP-3 responses were significantly (*p* = 0.040 and *p* = 0.007) different between running and jumping (COMP: jumping + 31%, running + 37%; MMP-3: jumping + 14%, running + 78%). Resistin increased only significantly (*p* < 0.001) after running, and Coll2-1 NO_2_ increased significantly (*p* = 0.001) only after jumping. Significant correlations between the biomarkers were detected. The relationships between individual serum biomarker concentrations may reflect the complex interactions between degrading enzymes and their substrates in ECM homeostasis.

## Introduction

Articular cartilage is a highly specialized connective tissue in synovial joints. It is responsible for a smooth and almost frictionless articulation between bones, as well as the transmission and distribution of joint contact forces. Because of its avascular nature, cartilage has only a very limited capacity for regeneration and self-repair. A certain extent of joint loading and mobilization is necessary to maintain cartilage homeostasis^1,2^. Moderate joint loading has been shown to initiate anabolic processes^3–5^. Immobilization as well as overloading, e.g., caused by malalignment, can induce an imbalance in articular cartilage metabolism, initiating catabolic pathways^6–8^.

Biochemical markers (biomarkers) can be used to monitor cartilage metabolism and could act as prognostic or diagnostic markers to detect changes in joint health^9^. Many of these biomarkers are essential components of the cartilage extracellular matrix (ECM). The measurement of cartilage biomarkers is based on the principle that osmotic and mechanical loading induce an efflux of proteolytically generated ECM fragments into the synovial fluid and subsequently out of the joint capsule. Thus, biomarker concentrations can be quantified by immunoassays in synovial fluid, blood and urine. Interestingly, biomarker concentrations are also used to explore the acute or long-term effect of exercise on articular cartilage health and metabolism^1,10^.

The most investigated serum cartilage biomarker is cartilage oligomeric matrix protein (COMP). COMP, which is also known as thrombospondin-5, is a pentameric glycoprotein consisting of five identical subunits. The C-terminal end of each monomer interacts with numerous ECM proteins, such as collagen II, collagen IX, matrilins and proteoglycans^11–14^. Further, COMP influences collagen secretion and fibrillogenesis, affecting the fibril formation rate^15^, arrangement^16^ and diameter^17^. Due to these interactions with other ECM proteins, COMP has a major role in the assembly and structure of the cartilage matrix, determining its mechanical properties. Based on these properties, COMP is widely studied with respect to acute loading^18^ and has been presented to be mechanosensitive^19–21^. Serum COMP levels have been shown to increase after 30 min of moderate running interventions by − 16% to + 39%^22–26^. However, no increase in serum concentration could be detected in response to other acute exercises, such as knee bends^22^ or repetitive lumbar flexion/extension^27^. This suggests that the acute serum COMP response depends on loading characteristics.

Other biomarkers of interest are proteolytic enzymes involved in the fragmentation of cartilage ECM components. Matrix metalloproteinases (MMPs) are a family of enzymes that vary in substrate specificity and their primary structure^28^. Certain MMP family members are involved in both ECM remodeling and pathological degradation. For example, MMP-3 (stromelysin) degrades most components of the ECM and activates other MMPs, such as MMP-9 (gelatinase B)^29^, which itself plays an important role in OA development^30^.

YKL-40 is another ECM protein that is involved in cartilage pathogenesis^31,32^. Even though the function of YKL-40 has not been identified in detail, as a glycosylase, it was shown to be generally involved in tissue remodeling and inflammatory processes^33^. YKL-40 levels are increased during joint inflammation, and resistin levels are also elevated by inflammatory stimuli^34,35^. Resistin is an adipokine initially associated with obesity and insulin resistance in rodents^36^.

The response of MMPs, YKL-40 and resistin concentrations to exercise has been investigated previously. Running a marathon resulted in increased serum concentrations of MMP-3 (+ 142%), YKL-40 (+ 56%) and resistin (+ 107%) immediately after the marathon compared to baseline values^37^. Calisthenic training resulted in significantly higher MMP-3 and MMP-9 concentrations immediately after training compared to pre-training values. In contrast, no increase in either biomarker was detected for the resistance training group, assuming that the level of circulating MMPs may depend on the mode of exercise^38^.

Type II collagen is a fundamental component of articular cartilage. The fragmentation of type II collagen occurs through the activity of collagenases and MMP-9. Coll2-1 is a specific peptide epitope located in the triple helix of the type II collagen molecules and a marker of cartilage degeneration^39^. The nitrated form of Coll2-1 is called Coll2-1 NO_2_ and reflects oxidative-related cartilage matrix degradation^40^. The serum concentrations of Coll2-1 (− 13%) and Coll2-1 NO_2_ (− 19%) decreased after a marathon^41^. Nagaoka et al.^42^ evaluated the cartilage collagen metabolism of athletes exposed to intense joint loading. They measured both type II collagen synthesis and the degradation biomarker in urine. The results indicated that type II collagen degradation is increased in response to endurance exercise with intense joint loading, especially in athletes with frequent jumping actions, such as volleyball, basketball and handball.

Although several studies have examined the effect of exercise on different biomarkers of cartilage metabolism, most analyses have not been performed systematically. Often, only one or a small number of ECM biomarkers are evaluated. Furthermore, in most studies, changes in biomarker levels were investigated only after one specific type of exercise. Therefore, it is largely unknown how biomarkers react to exercise regimes with different loading characteristics. Interlaboratory variability and minor variations in study protocols make it extremely difficult to compare results from studies using different interventions to better understand the role of loading characteristics and their impact on serum concentrations of soluble markers. Observing a wide range of ECM biomarkers at the same sampling time points would allow us to investigate correlations of different metabolic processes in the cartilage ECM network, as well as the interactions between the biomarkers.

Therefore, the purpose of the present study was to investigate 1) the acute response of seven biomarkers to two different exercise regimes (running and jumping) characterized by different loading magnitudes and frequencies and 2) the interaction of the selected biomarkers.

## Results

### Changes in biomarker concentrations in response to exercise

**Figure 1:**
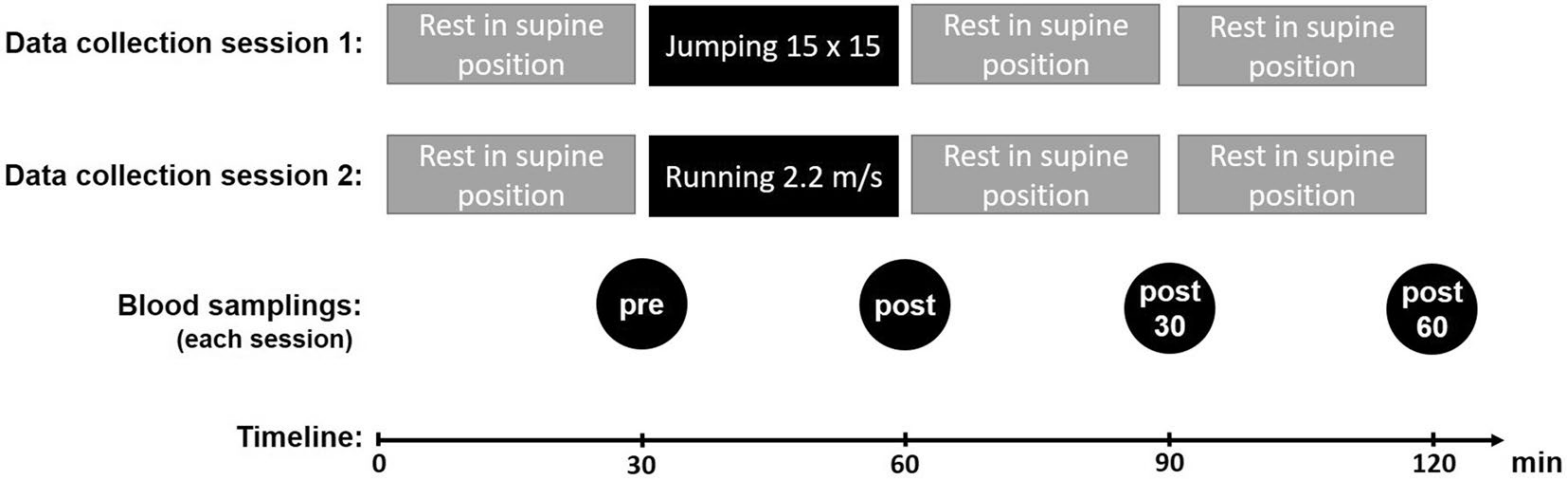
Study design of the two data collection sessions. The first data collection session included a 30 min jump exercise, and the second data collection session included 30 min of running. Both sessions were scheduled equally with four blood samplings every 30 min (“pre” directly before the exercise, “post” immediately after the exercise, “post30” 30 min after the exercise and “post60” 60 min after the exercise).

All fifteen participants (Table 1) completed both exercise protocols and donated all the required blood samples (Fig. 1). However, the serum COMP, Coll2-1 and Coll2-1 NO_2_ concentrations of one participant could not be analyzed at some time points because the collected serum volume was not enough, reducing the number to fourteen (N = 14) for these biomarkers.

**Table 1:** Demographic data of the subjects (N = 15). Data are presented as the mean (95% CI).

N	Age [years]	Body height [cm]	Body mass [kg]	BMI [kg/m^2^]	Sport activity per week [hours]
15	26 (24–29)	181 (177–185)	77.2 (74.0–80.5)	23.6 (22.7–24.6)	4.3 (2.5–6.1)

To examine the effect of the two different exercises (running and jumping) on the different biomarkers (COMP, YKL-40, MMP-3, MMP-9, resistin, Coll2-1 and Coll2-1 NO_2_), a two-way ANOVA was performed. Therefore, differences between the subsequent blood samplings were investigated as well as to the pre sampling within one exercise. In addition, differences between the exercises for the same time point were analyzed. No differences could be detected for the baseline blood sampling pre between running and jumping.

**Figure 2:**
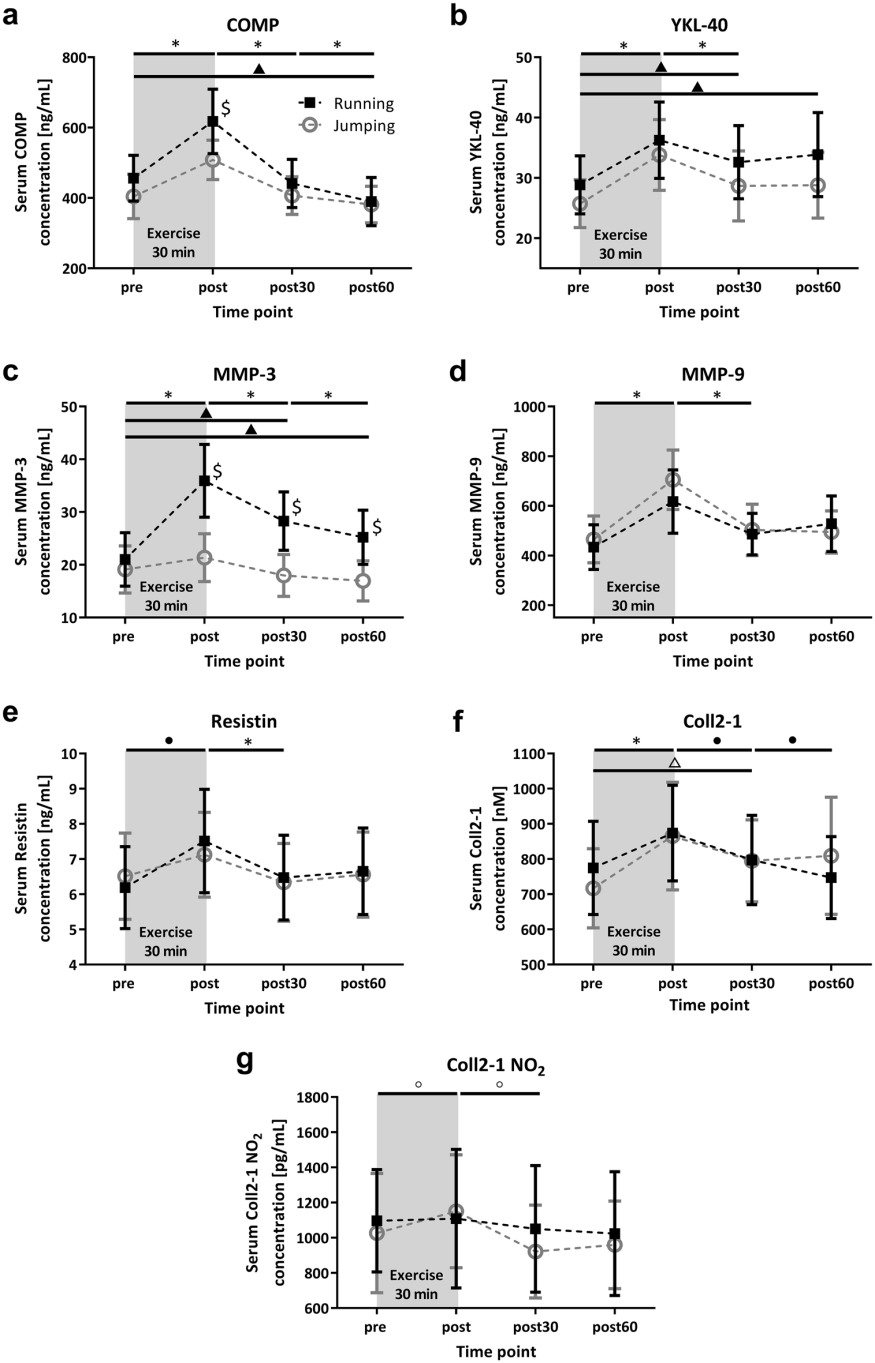
Mean (95% CI) serum (**a**) COMP (N = 14), (**b**) YKL-40 (N = 15), (**c**) MMP-3 (N = 15), (**d**) MMP-9 (N = 15), (**e**) resistin (N = 15), (**f**) Coll2-1 (N = 14) and (**g**) Coll2-1 NO_2_ (N = 14) concentrations before (pre), immediately (post), 30 min (post30), and 60 min (post60) after running and jumping exercise. *p < 0.05 significantly different from the preceding time point for both exercises. •*p* < 0.05 significantly different from preceding time point for the running exercise. °*p* < 0.05 significantly different from the preceding time point for jumping exercise. ^▲^*p* < 0.05 significantly different from the pre time point for the running exercise. ^△^*p* < 0.01 significantly different from the pre time point for jumping exercise. ^$^*p* < 0.05 indicates a significant difference between the running and jumping exercise groups at the same time point.

The serum COMP concentration increased significantly (*p* < 0.05) after both running and jumping exercises (pre compared to post, Fig. 2a, Tables S1 and S3). Following a significant (*p* < 0.05) decrease was detected 30 min after (post compared to post30) both exercise protocols. The COMP concentration decreased further (*p* < 0.05) for both exercise types until 60 min post exercise (post60). Prior to exercise, the serum COMP concentrations were variable but not significantly different between running and jumping. Interestingly, the serum COMP concentration immediately after exercise (post) was significantly higher (*p* < 0.05) in response to running than to jumping (Table S4). After running, an increase of + 36.8% (CI: 25.2–48.3) could be detected, while after jumping exercise, the increase was less pronounced at + 31.4% (CI: 11.5–51.3). In addition, the COMP concentration of the last blood drawing was significantly (*p* < 0.001) lower than that of the first blood drawing (pre compared to post) for the running exercise.

The serum YKL-40 concentration increased significantly (*p* < 0.01) from pre to post for both exercise regimes (Fig. 2b). The percent increase in the serum YKL-40 concentration was + 31.6% (CI: 21.4–41.9) for jumping and + 26.1% (CI: 19.0–33.1) for running. This was followed by a significant (*p* < 0.01) decrease from post to post30 for both exercise regimes, but the YKL-40 level stayed elevated for 30 to 60 min after the running exercise compared to the baseline level. Thus, significant differences from pre to post30 (*p* < 0.01) as well as from pre to post60 (*p* < 0.05) could be identified for the running exercise.

The serum MMP-3 concentration showed a significant (*p* < 0.05) increase from pre to post exercise and a significant (*p* < 0.05) decrease from post to post30 for both exercise regimes (Fig. 2c). However, the increase in running exercise + 77.5% (CI: 64.7–90.3) was much more pronounced than that for jumping exercise + 14.0% (CI: 7.9–20.2). A further significant (*p* < 0.05) decrease from post30 to post60 could be detected for both exercises. Significant differences (*p* < 0.001) between the running and jumping exercise were identified for all three time points after the exercise (post, post30 and post60), with higher values for the running exercise. The MMP-3 concentration remained alleviated 30–60 min after running, causing significant differences from pre to post30 (*p* < 0.001) and from pre to post60 (*p* < 0.05) for the running exercise.

For the serum MMP-9 concentration, a significant (*p* < 0.05) increase from pre to post exercise as well as a significant (*p* < 0.05) decrease from post to post30 could be found for both exercise regimes (Fig. 2d). The increase was + 46.8% (CI: 29.3–64.4) for running and + 59.7% (CI: 37.3–82.1) for jumping, but these increases were not significantly different between the exercise regimes.

The serum resistin concentration increased significantly (*p* < 0.001) only immediately after running exercise (+ 21.1%, CI: 14.8–27.4). From post to post30, a significant decrease (*p* < 0.01) was detected in the serum resistin concentration for both exercise regimes (Fig. 2e). The acute resistin response was not different between the running and jumping exercise.

Both exercise types induced a significant (*p* < 0.01) increase in the serum Coll2-1 concentration from pre to post exercise (running: + 13.7%, CI: 8.9–18.4; jumping: + 20.4%, CI: 10.3–30.5), but the subsequent decrease from post to post30 and post30 to post60 was only significant (*p* < 0.01) for the running exercise (Fig. 2f). For jumping exercise, the Coll2-1 concentration was still significantly (*p* < 0.01) higher 30 min after exercise than at the beginning (pre compared to post30).

Interestingly, the serum Coll2-1 NO_2_ concentration showed a different response to the two exercises; just for the jumping exercise, a significant response could be detected (Fig. 2g). The increase from pre to post (+ 17.9%, CI: 7.2–28.6) and the decrease from post to post30 was only significant (*p* < 0.01) for jumping exercise and not for running exercise.

All mentioned and graphically presented data are tabled with absolute and normalized values in Tables S1 and S2, as well as the *p*-values in Tables S3 and S4.

### Correlations between serum biomarker concentrations

Correlation analysis was performed with Spearman’s rank correlation coefficients (r_s_). The coefficients were calculated with the differences in the subsequent blood samplings for each participant and biomarker, representing the individual changes between the consecutive samplings. Due to power issues, the correlation analysis was performed together for the jumping and running exercise.

**Figure 3:**
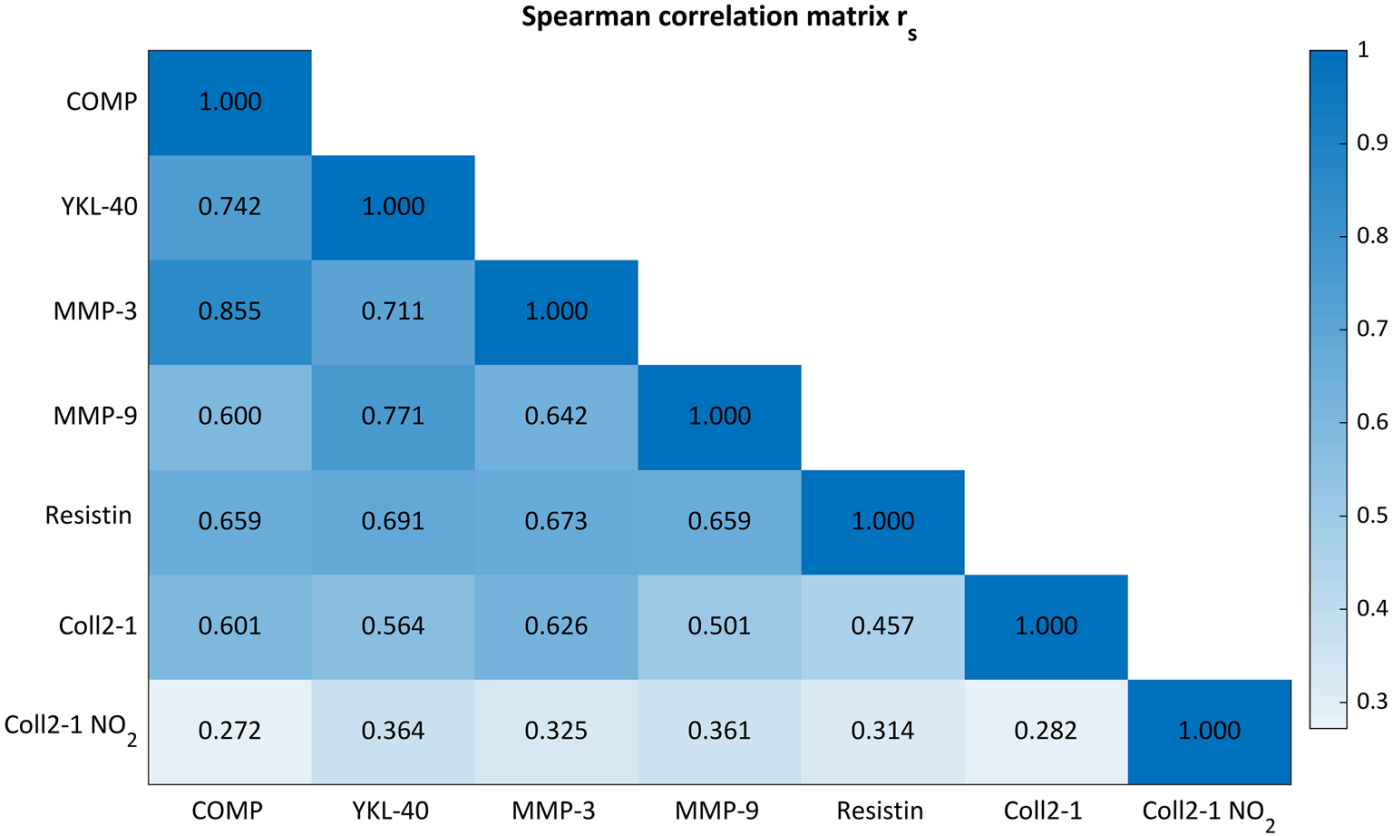
Spearman correlation matrix of absolute concentration changes of subsequent sampling time points. All presented coefficients are significant (*p* < 0.05).

**Figure 4:**
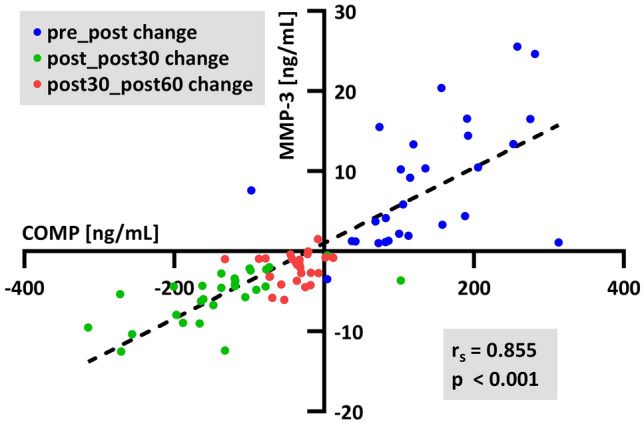
Spearman correlation of absolute concentration changes of subsequent sampling time points between COMP and MMP-3.

**Figure 5:**
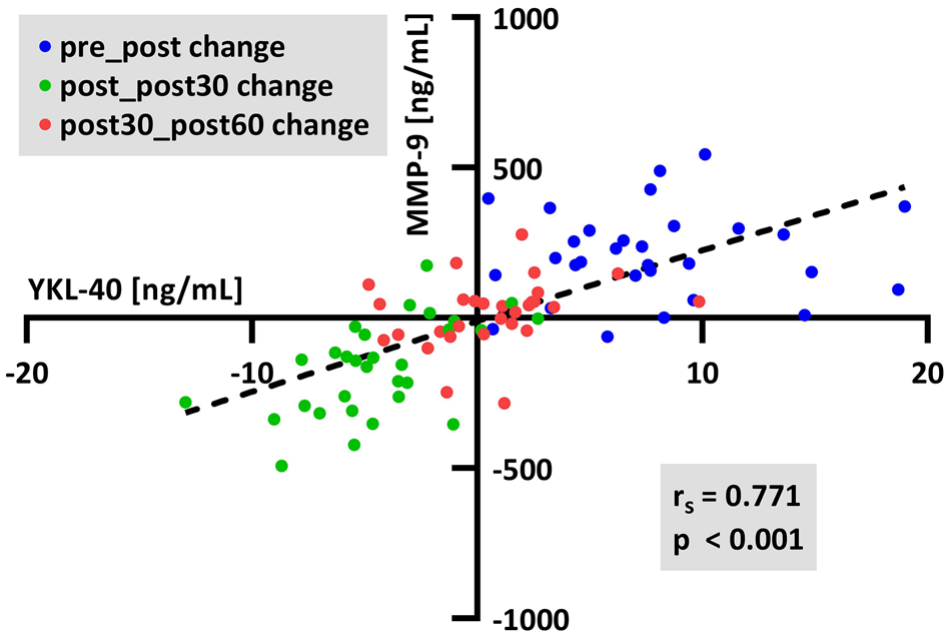
Spearman correlation of absolute concentration changes of subsequent sampling time points between YKL-40 and MMP-9.

Surprisingly, all calculated coefficients were significant. The correlation coefficients ranged between 0.27 and 0.85 (Fig. 3), describing a strength of correlation from “weak” to “very strong”. The strongest correlation was identified between COMP and MMP-3 (r_s_ = 0.855, *p* < 0.001, Fig. 4), and the second strongest was identified between YKL-40 and MMP-9 (r_s_ = 0.771, *p* < 0.001, Fig. 5). A clustering of the different changes can be identified. The change from pre to post is mostly positive (blue), while from post to post30 is mainly negative (green). The subsequent changes from post30 to post60 are approximately zero (red). For the correlation of COMP and MMP-3, the clusters are quite separated, but with diminishing correlation coefficients, the groups start to become cluttered.

## Discussion

In this study, a systematic evaluation of seven biochemical markers, related to cartilage metabolism, in response to two different mechanical loading exercises (jumping and running) was accomplished. The various biomarkers responded differently to the exercises, whereby the altered loading characteristics in particular seem to influence the biomarker response. In addition, the correlation analysis revealed some interesting interactions between the biomarkers.

The immediate increase and subsequent decrease in cartilage biomarker concentrations in response to acute exercise has been shown in previous studies^20,22,24,37^. However, the underlying mechanisms of this acute response are not completely understood. In pathological conditions such as OA, rheumatoid arthritis and acute joint injuries, the increase in cartilage biomarker concentrations is mainly interpreted as the progression of catabolic processes and cartilage degeneration. In contrast, an increase in cartilage biomarker levels induced by an acute exercise bout is generally assumed to indicate an increase in cartilage metabolism and associated remodeling processes^1,10^. However, correlations demonstrating a direct relation between loading characteristics and changes in biomarker concentration are missing. For example, no direct relation could be identified between the knee moment as an indicator of joint loading and an increase in cartilage biomarker concentrations^21,24,43^. This raises the question of whether an increase in serum biomarker concentrations might be the consequence of a general upregulation of the metabolic rate while exercising and not caused by local changes in articular cartilage metabolism.

However, there are also studies that did not detect an effect on serum biomarker concentrations after physical activity. For example, the biomarker COMP did not increase after performing repetitive lumbar flexion/extension tasks over 60 min, even though interleukin-6 and creatine kinase were significantly elevated^27^. Furthermore, the MMP-3 and MMP-9 serum concentrations were not elevated after acute resistance exercise^38^, and the serum COMP concentration did not increase after acute knee bending exercise or lymphatic drainage^22^. Taken together, not all these acute physical activities resulted in an increase in cartilage biomarker concentrations, even though the activities were demanding, and a general enhancement of the metabolic rate could be expected. However, they have in common that the exercise modes did not include high impact forces and that the forces applied on the lower extremities can be regarded as rather low.

Several studies indicate that cartilage metabolism is more sensitive to dynamic loading than to static loading^3–5^. This might also explain the observed difference in the extent of increase between jumping and running when specific biomarkers (COMP and MMP-3) were tested. For running exercise (~ 2422 impacts per leg), the number of impacts per leg was almost tenfold higher than for jumping exercise (~ 254 impacts per leg)^44^. In addition, between each jumping series, which lasted approximately 12 s, a resting period of approximately 75 s was included, and regeneration or compensation of the biomarker concentration may occur even during this relatively short resting period, resulting in a less measurable increase in serum concentration compared to running exercise. This hypothesis is supported by the study of Jayabalan et al.^45^, who observed a cumulative increase in serum COMP concentration comparing continuous walking for 45 min with intermittent walking for three times 15 min of walking separated by rest periods.

The decay behavior, especially after running exercise, illustrates an interesting course of response. For example, YKL-40 and MMP-3 demonstrated no decay behavior with significantly higher concentrations 30 and 60 min after running exercise compared to the starting value. The COMP level reached the initial concentration 30 min after the exercises. For the running exercise, an overshooting 60 min after exercise can be noted in comparison to the starting value. The decay behavior seems to be influenced by the loading characteristic of the exercise, with a faster return to baseline for the jumping exercise. Furthermore, decay is affected by the investigated biomarker itself; for example, COMP had a faster return to baseline, while YKL-40 and MMP-3 stayed elevated longer. Other influencing factors might be the duration of the exercise or participant characteristics, such as sex, age, weight and physical condition^1^.

Immediately after exercise, COMP and MMP-3 had significantly higher concentrations after running than after jumping. This might indicate that these biomarkers are highly mechanosensitive and particularly reinforced by the loading characteristics elicited by the running movement.

COMP is a substrate of both MMP-3 and MMP-9^46^, and for MMP family members, it has been shown that they can cleave COMP^47^. The correlation coefficient of MMP-9 and COMP demonstrated a strong relationship of the changes (r_s_ = 0.600, *p* < 0.001), and we identified an even stronger significant correlation for COMP with MMP-3 (r_s_ = 0.855, *p* < 0.001). Additionally, Mündermann et al.^20^ detected an association of approximately 30% (Wald Z = 3.476, *p* < 0.001) between changes in MMP-3 and COMP during an ultramarathon in 68% of runners.

Correlation analysis can be applied to elaborate a representative marker that reflects the dynamic response of several biomarkers. Therefore, in subsequent studies and/or as a diagnostic tool, it might be sufficient to reduce the analysis of these representative biomarkers. In addition, the correlation analysis gives further insight into the relationships between biomarkers and thereby draws conclusions about the underlying protein interactions. Similar dynamic behavior and high correlations might be a reflection of a dependency on the same signaling pathways, for example, the MAPK/ERK pathway (mitogen-activated protein kinases, originally called ERK, extracellular signal-regulated kinases), which is involved in mechanotransduction. COMP, MMP-3 and YKL-40 reacted notably mechanosensitive to the exercises applied here and presented higher correlations between each other, perhaps due to overriding signaling cascades.

The graphical representation of the correlation analysis demonstrates a grouping of the changes between the measurement time points. The first change is mainly positive, followed by a negative change and a steady state with almost no change. As many more participants display a similar reaction on the exercises and for each biomarker the higher are the correlation coefficients between the biomarker and the discriminatory power of the clusters. This might suggest that mechanosensitive biomarkers in particular follow a specific behavior and thus have higher correlation coefficients and discriminatory power.

Some of the detected correlations could be explained by direct interactions of the corresponding ECM proteins. To further strengthen the hypothesis that biomarker concentration could provide additional insight into ECM composition and structure, more biomarkers have to be measured, and multiple regression analysis/computational modeling^48^ needs to be performed.

The present study also has some constraints. When measuring biomarker concentrations in serum samples, the origin of the analyzed protein remains unknown. The measured proteins can also be extruded from tissues other than cartilage, such as tendons, meniscus, synovium and arterial walls^49^. Some of the biomarkers have also been investigated as prognostic markers and potential therapeutic targets in cancer research^50,51^. Various proteins are regulated by the same pathways that are also activated in OA, such as PI3K/AKT/mTOR (phosphatidylinositol-3-kinase/protein kinase B/mammalian target of rapamycin)^52^. Nevertheless, all biomarkers examined here were investigated mainly due to OA^49,53^ and articular joint metabolism in general^1,54^. The measured biomarker concentration is based on a correlation between biomarker concentrations in serum and synovial fluid^55–58^. Due to the standardization of the sessions, detected differences between the exercises are likely due to the varying loading conditions onto the lower extremities. In addition, the number of participants is low and thus influences the power of the study; therefore, a crossover design was chosen to minimize the effects of interindividual variations. In future studies, biomarkers for assessing the general metabolic rate should be included.

In conclusion, we performed a systematic and comprehensive analysis of seven serum biomarkers in response to two frequently used exercise interventions (jumping and running). We could show exercise-specific changes in serum biomarker concentrations and significant correlations between distinct biomarkers. The increase in various ECM proteins was more pronounced after 30 min of running than after 30 min of jumping, suggesting a strong dependence on the loading characteristics. Correlations in serum biomarker concentrations indicate either a functional interaction between distinct ECM proteins or their role as enzyme–substrate pairs. On the one hand, our study provides better insight into the potential of biochemical markers for the evaluation of the cartilage status in healthy individuals but also patients suffering from diseases such as osteoarthritis. On the other hand, a better understanding of the effects of exercise on cartilage metabolism and interacting proteins will allow to develop better training protocols and to monitor treatment effects.

## Methods

### Subjects

Fifteen healthy male adults participated in this study (Table 1). The inclusion criteria were an age between 20 and 35 years at the time of recruitment. The participants were healthy and physically active individuals, performing two to three training sessions per week. To avoid the influences of previous medical conditions, we excluded participants with acute or chronic injuries of the lower extremities, musculoskeletal disorders and surgical interventions of the knee joint. We recorded the full history of lower extremity injuries. The participants were instructed to minimize physical activities 48 h prior to the experimental days, which obstructed all sporting activities, but normal daily activities, such as walking and stair climbing, were allowed. In addition, the participants were advised to keep the same daily routine and to eat the same on measurement days. The study was conducted in accordance with the Declaration of Helsinki, ratified by the local ethics committee of the North Rhine Medical Association (Aerztekammer Nordrhein) and registered in the German clinical trials register (DRKS-ID: DRKS00014001). Prior to the start of the experiment, we received written informed consent from all participants.

### Study design

Two different 30 min exercise regimes were conducted during two data collection sessions scheduled on two different days (Fig. 1). To avoid diurnal variations the participants were asked to be present at the same time of day at the laboratory. There was at least one week of rest between the two sessions to reduce potential carryover effects. During session 1, the participants performed reactive jumps. In session 2, the participants were running on a treadmill. Except for the 30 min of exercising, both data collection sessions were structured identically.

The participants were familiarized with the correct jumping performance at least three days before data collection. The correct jumping movement is characterized by short ground contact times, high peak ground reaction forces and a high preactivity of the leg extensor muscles^59^.

### Data collection sessions

After the subject arrived at the laboratory, an indwelling venous catheter (Vasofix® Sadty, B. Braun, Melsungen, Germany) was inserted into the cubital fossae of the right or left arm. First, the volunteers performed a short mobilization exercise consisting of 10 submaximal knee bends and 10 submaximal tiptoe raisings. After that, a 30 min resting period started to minimize the potential influences of preceding physical activity on the biomarker concentration. The subjects had to lie in the supine position on a stretcher. At the end of the resting period, the first blood sample (pre) was taken (Fig. 1). Subsequently, the subjects completed 15 × 15 reactive jumps or a running session on a treadmill (Treadmetrix, Park City, UT, USA) at a set velocity of 2.2 m/s, and both sessions were completed within 30 min. Immediately after exercise, the subjects rested again on the stretcher, and follow-up blood samples were collected immediately (post), 30 min (post30) and 60 min (post60) after the end of the exercise.

### Blood sampling and biomarker analysis

Blood samples (each 8 ml) were drawn from the antecubital vein into two vacutainers (BD Vacutainer® SSTTM II Advance, Becton Dickinson and Co., Franklin Lakes, NJ, USA) and were then allowed to clot for 30 min. After centrifugation (1500 g for 10 min), serum samples were aliquoted and stored at − 80 °C until analysis.

Throughout the biomarker analysis, the investigators were blinded to the samples, and all samples were analyzed in duplicate and in random order. All seven biomarkers were analyzed according to the manufacturer’s specifications and all data were above the detection limit. The biomarker COMP was investigated using ELISA from BioVendor (BioVendor, Brno, Czech Republic; detection sensitivity: < 0.4 ng/mL, intra-assay variability: 8.0%). In addition, YKL-40 (MicroVueTM Bone, Quidel, San Diego, USA; detection sensitivity: < 5.4 ng/mL, intra-assay variability: 7.0%), MMP-3 (Quantikine®, Bio-Techne, R&D Systems, Minneapolis, USA; detection sensitivity: from 0.002 to 0.045 ng/mL, intra-assay variability: 8.6%), MMP-9 (Quantikine®, Bio-Techne, R&D Systems, Minneapolis, USA; detection sensitivity: < 0.156 ng/mL, intra-assay variability: 7.9%), and resistin (Quantikine®, Bio-Techne, R&D Systems, Minneapolis, USA; detection sensitivity: from 0.010 to 0.055 ng/mL, intra-assay variability: 9.2%) were measured. Furthermore, concentrations of Coll2-1 (Artialis SA, Liege, Belgium; detection sensitivity: < 21.83 nM, intra-assay variability: 21.0%) and of Coll2-1 NO_2_ (Artialis SA, Liege, Belgium; detection sensitivity: < 38 pg/mL, intra-assay variability: 13.0%) were determined. Technical performance data were specified by the manufacturers. To minimize the interassay variability, all samples of one participant were measured on the same ELISA plate. Serum dilution factors were as following: COMP 50-fold dilution, YKL-40 no dilution, MMP-3 tenfold dilution, MMP-9 100-fold dilution, resistin fivefold dilution, Coll2-1 and Coll2-1 NO_2_ threefold dilutions.

### Statistical analysis

The statistical analysis was performed using MATLAB R2019b (MathWork, Natrick, MA, USA). Normal distribution was tested with the Shapiro–Wilk test. The homoscedasticity of variance for the repeated measures was checked using Mauchly’s sphericity test; if variance homogeneity was not fulfilled, we performed a Greenhouse–Geisser correction. A two-way analysis of variance (ANOVA) with repeated measurements and Tukey–Kramer test for post hoc analysis were performed to detect differences in the serum biomarker levels between the exercises (jumping and running) and the consecutive blood samplings (pre, post, post30 and post60) as well as to the pre blood sampling. The correlation between different biomarkers was analyzed using the Spearman rank correlation coefficient (r_s_); therefore, the difference in the consecutive blood samples was calculated (post minus pre, post30 minus post and post60 minus post30). The correlation analysis for the jumping and running exercise was performed together. All variables are described as the mean values, and the confidence interval was set at 95% (95% Cl: lower limit, upper limit). We performed all statistical tests on absolute values, and only for comprehensible reasons were the biomarker data normalized to baseline values measured at the blood sampling time point pre. The level of significance was set at *α* < 0.05.

## Supplementary Information

Supplementary Information.
