# Author Correction: Relationship between different serum cartilage biomarkers in the acute response to running and jumping in healthy male individuals

**DOI:** 10.1038/s41598-022-11194-9

**Published:** 2022-04-25

**Authors:** Maren Dreiner, Tobias Munk, Frank Zaucke, Anna-Maria Liphardt, Anja Niehoff

**Affiliations:** 1 Institute of Biomechanics and Orthopaedics, German Sport University Cologne, Am Sportpark Müngersdorf 6, 50933 Cologne, Germany; 2 Dr. Rolf M. Schwiete Research Unit for Osteoarthritis, Department of Orthopaedics (Friedrichsheim), University Hospital Frankfurt, Goethe University, Frankfurt, Germany; 3 Department of Internal Medicine 3 - Rheumatology and Immunology, Universitätsklinikum Erlangen and Friedrich-Alexander University Erlangen-Nürnberg, Erlangen, Germany; 4 Faculty of Medicine, Cologne Center for Musculoskeletal Biomechanics (CCMB), University of Cologne, Cologne, Germany

Correction to: *Scientific Reports* 10.1038/s41598-022-10310-z, Published online 19 April 2022

The original version of this Article contained errors in the spelling of the authors Maren Dreiner, Tobias Munk, Frank Zaucke, Anna-Maria Liphardt & Anja Niehoff which were incorrectly given as Dreiner Maren, Munk Tobias, Zaucke Frank, Liphardt Anna-Maria & Niehoff Anja respectively.

The original Article has been corrected.

