# Supplementary material for: Relationship between different serum cartilage biomarkers in the acute response to running and jumping in healthy male individuals
Source: Sci Rep. 2022 Apr 19;12:6434. doi: 10.1038/s41598-022-10310-z (PMC9018733; doi:10.1038/s41598-022-10310-z)
Supplement: Supplementary file 1 — Supplementary Information. [file 41598_2022_10310_MOESM1_ESM.docx]

**Relationship between different serum cartilage biomarkers in the acute response to running and jumping in healthy male individuals**

Dreiner, Maren^1^; Munk, Tobias^1^; Zaucke, Frank^2^; Liphardt, Anna-Maria^3^; Niehoff, Anja^1,4*^

^1^Institute of Biomechanics and Orthopaedics, German Sport University Cologne, Cologne, Germany

^2^Dr. Rolf M. Schwiete Research Unit for Osteoarthritis, Department of Orthopaedics (Friedrichsheim), University Hospital Frankfurt, Goethe University, Frankfurt, Germany

^3^Department of Internal Medicine 3 - Rheumatology and Immunology, Universitätsklinikum Erlangen, Friedrich-Alexander University Erlangen-Nürnberg, Erlangen, Germany.

^4^Cologne Center for Musculoskeletal Biomechanics (CCMB), Faculty of Medicine, University of Cologne, Cologne, Germany

*Anja Niehoff, Ph.D., Institute of Biomechanics and Orthopaedics, German Sport University Cologne, Am Sportpark Müngersdorf 6, 50933 Köln, Germany, E-mail: niehoff@dshs-koeln.de

Table S1: Mean (95% CI) serum biomarker concentrations before (pre), immediately (post), 30 min (post30), and 60 min (post60) after the running and jumping exercise.

| **Biomarker** | **N** | **Exercise** | **pre** | | | **post** | | | **post30** | | | **post60** | | |
| --- | --- | --- | --- | --- | --- | --- | --- | --- | --- | --- | --- | --- | --- | --- |
|  |  |  | **Mean** | **95% CI** | | **mean** | **95% CI** | | **mean** | **95% CI** | | **mean** | **95% CI** | |
| COMP [ng/mL] | 14 | Running | 456.2 | 391.5 | 521.0 | 617.8*^$^ | 526.2 | 709.5 | 441.0* | 372.5 | 509.6 | 389.9*° | 321.2 | 458.6 |
|  |  | Jumping | 404.4 | 341.2 | 467.6 | 508.2*^$^ | 452.3 | 564.0 | 406.5* | 352.8 | 460.3 | 381.1* | 328.9 | 433.2 |
| YKL-40 [ng/mL] | 15 | Running | 28.8 | 24.0 | 33.6 | 36.3* | 29.9 | 42.6 | 32.6*° | 26.5 | 38.7 | 33.9° | 26.9 | 40.8 |
|  |  | Jumping | 25.7 | 21.8 | 29.7 | 33.8* | 27.9 | 39.7 | 28.7* | 22.9 | 34.5 | 28.8 | 23.3 | 34.3 |
| MMP-3 [ng/mL] | 15 | Running | 21.0 | 16.0 | 26.1 | 35.9*^$^ | 29.0 | 42.8 | 28.3*°^$^ | 22.7 | 33.8 | 25.2*°^$^ | 20.1 | 30.4 |
|  |  | Jumping | 19.1 | 14.6 | 23.6 | 21.3*^$^ | 16.8 | 25.9 | 18.0*^$^ | 14.0 | 22.0 | 16.9*^$^ | 13.2 | 20.7 |
| MMP-9 [ng/mL] | 15 | Running | 433.4 | 342.9 | 523.9 | 617.0* | 489.1 | 744.8 | 486.2* | 402.8 | 569.6 | 528.2 | 415.9 | 640.4 |
|  |  | Jumping | 464.9 | 370.9 | 558.8 | 705.1* | 585.0 | 825.2 | 503.3* | 399.7 | 607.0 | 494.6 | 409.4 | 579.8 |
| Resistin [ng/mL] | 15 | Running | 6.2 | 5.0 | 7.4 | 7.5* | 6.0 | 9.0 | 6.5* | 5.3 | 7.7 | 6.7 | 5.4 | 7.9 |
|  |  | Jumping | 6.5 | 5.3 | 7.7 | 7.1 | 5.9 | 8.3 | 6.3* | 5.2 | 7.5 | 6.6 | 5.3 | 7.8 |
| Coll2-1 [nM] | 14 | Running | 774.7 | 642.0 | 907.5 | 873.6* | 737.5 | 1009.7 | 797.4* | 670.5 | 924.4 | 747.4* | 631.0 | 863.8 |
|  |  | Jumping | 716.9 | 604.3 | 829.6 | 865.2* | 712.2 | 1018.3 | 794.6° | 678.1 | 911.1 | 809.3 | 642.9 | 975.6 |
| Coll2-1 NO_2_ [pg/mL] | 14 | Running | 1097.0 | 805.9 | 1388.0 | 1108.4 | 714.6 | 1502.1 | 1050.1 | 690.0 | 1410.2 | 1023.1 | 671.1 | 1375.1 |
|  |  | Jumping | 1026.9 | 687.6 | 1366.2 | 1150.7* | 829.8 | 1471.6 | 921.6* | 657.4 | 1185.8 | 959.5 | 710.3 | 1208.7 |

*p < 0.05 significantly different to the previous blood sampling (same exercise)

°p < 0.05 significantly different to the pre blood sampling (same exercise)

^$^p < 0.05 significantly different between running and jumping exercise (same blood sampling)

Table S2: Mean (95% CI) serum biomarker concentrations before (pre) and immediately (post), 30 min (post30) and 60 min (post60) after the running and jumping exercise. The serum biomarker concentrations were normalized to the blood sampling time point pre (100%).

| **Biomarker** | **N** | **Exercise** | **pre** | **post** | | | **post30** | | | **post60** | | |
| --- | --- | --- | --- | --- | --- | --- | --- | --- | --- | --- | --- | --- |
|  |  |  | **mean** | **mean** | **95% CI** | | **mean** | **95% CI** | | **mean** | **95% CI** | |
| COMP | 14 | Running | **100.0** | **136.8** | 125.2 | 148.3 | **96.1** | 93.0 | 99.3 | **84.5** | 79.4 | 89.6 |
|  |  | Jumping | **100.0** | **131.4** | 111.5 | 151.3 | **106.1** | 82.7 | 129.5 | **98.1** | 79.5 | 116.8 |
| YKL-40 | 15 | Running | **100.0** | **126.1** | 119.0 | 133.1 | **112.7** | 106.8 | 118.6 | **116.1** | 106.3 | 126.0 |
|  |  | Jumping | **100.0** | **131.6** | 121.4 | 141.9 | **110.9** | 101.0 | 120.7 | **111.9** | 102.2 | 121.7 |
| MMP-3 | 15 | Running | **100.0** | **177.5** | 164.7 | 190.3 | **139.4** | 129.9 | 148.9 | **123.9** | 114.7 | 133.1 |
|  |  | Jumping | **100.0** | **114.0** | 107.9 | 120.2 | **95.7** | 90.6 | 100.8 | **89.7** | 84.7 | 94.7 |
| MMP-9 | 15 | Running | **100.0** | **146.8** | 129.3 | 164.4 | **118.6** | 101.9 | 135.2 | **126.3** | 106.4 | 146.1 |
|  |  | Jumping | **100.0** | **159.7** | 137.3 | 182.1 | **114.1** | 89.4 | 138.7 | **111.8** | 95.1 | 128.5 |
| Resistin | 15 | Running | **100.0** | **121.1** | 114.8 | 127.4 | **105.9** | 98.4 | 113.3 | **110.6** | 96.3 | 124.9 |
|  |  | Jumping | **100.0** | **111.2** | 102.0 | 120.5 | **98.9** | 91.0 | 106.8 | **101.6** | 94.0 | 109.2 |
| Coll2-1 | 14 | Running | **100.0** | **113.7** | 108.9 | 118.4 | **103.5** | 98.7 | 108.3 | **97.2** | 92.5 | 101.8 |
|  |  | Jumping | **100.0** | **120.4** | 110.3 | 130.5 | **111.4** | 103.9 | 118.9 | **116.1** | 90.7 | 141.5 |
| Coll2-1 NO_2_ | 14 | Running | **100.0** | **98.8** | 88.1 | 109.4 | **94.6** | 82.3 | 106.9 | **93.5** | 76.2 | 110.7 |
|  |  | Jumping | **100.0** | **117.9** | 107.2 | 128.6 | **95.1** | 84.8 | 105.3 | **100.6** | 87.9 | 113.4 |

Table S3: P-values (two-way ANOVA with post-hoc test: Tukey-Kramer) for the comparisons of biomarker concentrations at different time points after the same exercise. Values before exercise (pre) were compared with all other sampling time points (immediately (post), 30 min (post30), and 60 min (post60) after exercise). In addition, values at each time point were compared with those at the preceding time point. Bold: p < 0.05 (statistically significant).

| **Biomarker** | **N** | **Exercise** | **Pre-post** | **pre-post30** | **pre-post60** | **post-post30** | **post30-post60** |
| --- | --- | --- | --- | --- | --- | --- | --- |
| COMP | 14 | Running | **0.0008** | 0.2957 | **0.0001** | **0.0003** | **0.0014** |
|  |  | Jumping | **0.0037** | 1.0000 | 0.9500 | **6.41E-06** | **0.0287** |
| YKL-40 | 15 | Running | **1.92E-05** | **0.0046** | **0.0128** | **0.0019** | 0.5513 |
|  |  | Jumping | **0.0004** | 0.2634 | 0.1497 | **0.0002** | 0.9975 |
| MMP-3 | 15 | Running | **8.33E-07** | **0.0001** | **0.0447** | **1.14E-06** | **0.0001** |
|  |  | Jumping | **0.0255** | 0.4888 | 0.0945 | **4.37E-06** | **0.0198** |
| MMP-9 | 15 | Running | **0.0006** | 0.2765 | 0.0958 | **0.0284** | 0.3611 |
|  |  | Jumping | **0.0003** | 0.8313 | 0.8123 | **0.0010** | 0.9928 |
| Resistin | 15 | Running | **0.0001** | 0.3291 | 0.2314 | **0.0034** | 0.7536 |
|  |  | Jumping | 0.1817 | 0.8994 | 0.9987 | **0.0010** | 0.5054 |
| Coll2-1 | 14 | Running | **0.0002** | 0.4968 | 0.4835 | **0.0003** | **0.0058** |
|  |  | Jumping | **0.0041** | **0.0042** | 0.6110 | 0.1564 | 0.9966 |
| Coll2-1 NO_2_ | 14 | Running | 0.9984 | 0.9081 | 0.8285 | 0.4121 | 0.7955 |
|  |  | Jumping | **0.0014** | 0.1723 | 0.6801 | **0.0006** | 0.5316 |

Table S4: P-values (two-way ANOVA with post-hoc test: Tukey-Kramer) for the comparison between running and jumping at the same sampling time point. Only listed biomarkers reached significance level (p < 0.05) in the interaction factor of the two-way repeated ANOVA. Bold: p < 0.05 (statistically significant).

| **Biomarker** | **N** | **pre-pre** | **post-post** | **post30-post30** | **post60-post60** |
| --- | --- | --- | --- | --- | --- |
| COMP | 14 | 0.6343 | **0.0404** | 0.6430 | 0.9998 |
| MMP-3 | 15 | 0.5803 | **2.81E-06** | **3.52E-06** | **6.48E-06** |
